# Standardized Extraction Techniques for Meat Analysis with the Electronic Tongue: A Case Study of Poultry and Red Meat Adulteration

**DOI:** 10.3390/s21020481

**Published:** 2021-01-12

**Authors:** John-Lewis Zinia Zaukuu, Zoltan Gillay, Zoltan Kovacs

**Affiliations:** Department of Measurement and Process Control, Faculty of Food Science, Szent István University, H-1118 Budapest, Hungary; gillay.zoltan@szie.hu (Z.G.); kovacs.zoltan.food@szie.hu (Z.K.)

**Keywords:** sensors, adulteration, fraud, chemometrics, prediction, methodology

## Abstract

The electronic tongue (e-tongue) is an advanced sensor-based device capable of detecting low concentration differences in solutions. It could have unparalleled advantages for meat quality control, but the challenges of standardized meat extraction methods represent a backdrop that has led to its scanty application in the meat industry. This study aimed to determine the optimal dilution level of meat extract for e-tongue evaluations and also to develop three standardized meat extraction methods. For practicality, the developed methods were applied to detect low levels of meat adulteration using beef and pork mixtures and turkey and chicken mixtures as case studies. Dilution factor of 1% *w*/*v* of liquid meat extract was determined to be the optimum for discriminating 1% *w*/*w*, 3% *w*/*w*, 5% *w*/*w*, 10% *w*/*w*, and 20% *w*/*w* chicken in turkey and pork in beef with linear discriminant analysis accuracies (LDA) of 78.13% (recognition) and 64.73% (validation). Even higher LDA accuracies of 89.62% (recognition) and 68.77% (validation) were achieved for discriminating 1% *w*/*w*, 3% *w*/*w*, 5% *w*/*w*, 10% *w*/*w*, and 20% *w*/*w* of pork in beef. Partial least square models could predict both sets of meat mixtures with good accuracies. Extraction by cooking was the best method for discriminating meat mixtures and can be applied for meat quality evaluations with the e-tongue.

## 1. Introduction

Meat is a central part of diets around the world and is considered as a primary source of protein across the globe. The world population increased by almost 4 billion in the last 50 years (128%) while the global average meat consumption per capita increased by 75% [1]. Demand for proteins from plant-based sources has remained stable over time but the same cannot be said for proteins from animal sources. There has been a sharp increase with animal products now accounting for 58% of protein availability per capita/day [2]. This implies that the global meat consumption and production almost quadrupled. Debates about meat production and consumption are often complex and controversial [3] but, the same can also be said about meat quality control. Meat quality is a rather complex concept, which includes different microbiological, physicochemical, and biochemical attributes [4] which can all be tampered with for financial gains. According to literature, protein from non-plant sources account for close to 30% of calories in the European Union (EU) and can be represented as 28 g of protein/capita daily [2]. Controlling meat quality is therefore of paramount importance because meat adulteration or misrepresentation can lead to consumer distrust in the meat value chain which can impact economic revenues. Misrepresentation of meat could equally also have bad implications from religious and moral perspectives as people have different preferences of meat they wish to consume.

Primarily, meat from animal sources can be classified into red meat and poultry [5]. Red meat refers to meat that often shows a red appearance. The most common among these are lamb also, sometimes referred to as mutton, pork, veal and beef. Red meat reportedly, is a good source some important nutrients [6] but may also, controversially, trigger coronary issues when certain processing methods are taken into consideration [3]. Poultry mainly encompasses birds in the fowl category among which, the common ones are turkey, chicken, geese and duck. For reasons such as nutritional quality and consumer preference or the part of animal that is being sold, the prices all these meat types can vary. For instance, beef is often regarded as more expensive red meat. In some Hungarian markets (central Europe), minced beef meat was sold at a higher price of 2898 HUF/kg (equivalent of 9.01 euro/kg) in comparison to meat pork which was sold at 1698HUF/kg (equivalent of 4.69 euro/kg). Minced turkey breast was also sold at a higher of 998 HUF/kg (equivalent of 2.76 euro/kg) in comparison to chicken breast which was sold at 499 HUF/kg (equivalent of 1.38 euro/kg). It is important to note that, some minced meat products already exist in many markets across the globe and can contain a mix of both high-priced and low-priced meat. This is acceptable so long as it is evident in the labelling of the product. What is unacceptable is mixing the high-priced meat with the low-priced meat during mincing and representing it as a 100% version of the expensive one, or falsely stating the applied mixing ratio. This type of adulteration be done at varying concentration levels of meat types depending on the meat type and market demand. Concentration levels with increasing scales of 5% *w*/*w* [7], 10% *w*/*w* [8], and 20% *w*/*w* [9] of meat adulteration are among the most common in literature.

For detecting such adulteration and tampering, many studies have explored different techniques. Common examples are mass spectrometry [10], polymerase chain reaction (PCR) [11,12] and enzyme-linked immunosorbent assay (ELISA) [13]. Due to cost, accuracy, reliability, less sophistication, and speed of the analytical process, other methods such as spectroscopy [7,14,15,16,17] and machine learning-enabled smart sensor systems [18], such as the electronic nose (e-nose) [8,9,19,20] and electronic tongue [19], have become more preferred methods for detecting meat adulteration. Both spectroscopy and e-nose are non-invasive techniques but spectroscopy operates on the principle of how the meat sample reacts with electromagnetic waves in a defined wavelength whereas, e-nose operates on the principle assessing odor signatures from the meat sample through high sensitivity sensors [20]. The electronic tongue (e-tongue) is another high sensitivity sensor based instrument capable of discriminating food products through pattern recognition [21] and has been used for both quantitative and qualitative environmental monitoring [22], pharmaceutical [23] and food analysis [22,24,25]. Like spectroscopy and e-nose, e-tongue provides rapid analytical outputs, affordable, easy to use and poses no risk to the user, unlike certain cases with sensory analysis [5]. Unlike spectroscopy and e-nose however, e-tongue is equipped with high sensitivity sensors that measure taste compounds/dissolved components. A detailed head-to-head comparison of some of these instruments and their applications have been reported [5,9,20,26] but this current study will focus solely on the e- tongue. Ideally, the e-tongue is better suited for liquid samples and combination with chemometric techniques, it has been used to monitor the quality of coffee [27], wine [24,28,29], fruit juice [24], oils [25,30,31], tea [32], and recently in some semi-solid foods like tomato concentrate [33]. With mathematical correction methods [28,29,32,34], signals from the e-tongue sensors can be further optimized to compensate for issues of drift that could arise from environmental factors, such as temperature, relative humidity, etc., or sensor aging [5,20,28].

E-tongue has also been scantly applied in the meat industry to monitor physical–chemical and microbiological changes in pork meat during storage [35], the impact of curing agents in meat [36] and even detect ammonia and putrescine in beef products [37]. However, nothing was mentioned about the sample extraction method in all the studies regarding the application of e-tongue for meat quality control. In fact, there is no clearly defined sample preparation method for meat analysis with the e-tongue. A standardized sample preparation is necessary because, a less effective method can decrease the sensitivity of the sensors [28] which, can negatively affect the results. Sensor signals from e-tongue can generally be correlated with descriptive sensory evaluations and other analytical methods to monitor or predict organoleptic properties in food [38], but unlike sensory evaluation where subjective or other analytical methods are targeted, the e-tongue principle is non-subjective and can be targeted or non-targeted [39]. Many e-tongues have been reported to have partial, selectivity and sensitivity to different ionic compounds in solution [5], all the properties of the analyte come into play in e-tongue non-targeted analysis. This emphasizes the need to explore different extraction methods because different extraction methods may result in different ionic compounds in the meat extract which, can influence the outcomes of e-tongue analyses.

This study aimed to determine the optimal dilution level of meat extract for e-tongue evaluation which currently remains a gap and also to develop three standardized sample preparation methods for meat analysis with the e-tongue as there is currently no standardized method and very limited application of the e-tongue for meat evaluations. For practicality, the developed methods were applied to detect low levels of meat adulteration using red meat (beef and pork adulteration) and poultry (turkey and chicken adulteration) as case studies. For purposes of this study, meat adulteration implies mixing of cheap minced meat with expensive meat at different concentrations.

## 2. Materials and Methods

### 2.1. Determination of Optimal Dilution

Fresh turkey and chicken breast were purchased from reputable supermarkets in Budapest, Hungary and transported to the laboratory. The meat samples were separately minced and artificially adulterated to four adulteration levels: 100% 97%, 95% and 90% *w*/*w* of turkey to have a total of 20 g per sample (meat mixture concentration). The concentration levels for this study were determined based on the commonly reported ranges in literature [7,8,9] but the extra lower level of 97% *w*/*w* turkey (3% *w*/*w* of chicken in turkey) was included to study the feasibility of e-tongue in discriminating lower concentrations than those reported in literature.

Each sample was extracted by transferring into a 200 mL volumetric flask and filled up to volume with distilled water then homogenized and filtered using a wire mesh filter (20 mesh) to obtain the stock filtrate. Three different dilution levels were prepared from the adulterated meat extracts (stock filtrates) as shown in Table 1. For dilution level 1, dilution level 2, and dilution level 3, 5 mL, 10 mL, and 20 mL from their respective filtrates were pipetted into separate 100 mL flasks, homogenized, and filled up to volume with distilled water before transferring into 100 mL glass beakers for e-tongue analysis.

### 2.2. Determination of Optimal Extraction Method

Three different methods were tested to determine the optimal extraction method for meat measurement with the e-tongue: raw meat extraction with distilled water, meat extraction by cooking with distilled water and Frozen meat extraction with distilled water. All the tested methods fall within the sustainable green technology category of the Sustainable Development Goals Index (SGDI) [40], which supports the exploration of non-chemical related analysis for environmental sustainability.

For each method, the determined optimum dilution level in the first experiment was applied to discriminate wider ranges of adulteration in poultry (turkey and chicken adulteration) and red meat (beef and pork). The fresh raw meat samples, were purchased from reputable supermarkets in Budapest, separately minced and artificially adulterated to five different adulteration levels by mixing them to have a total of 20 g per sample as shown in Table 2 for turkey chicken adulteration (T means turkey) and in Table 3 for beef and pork adulteration (B means beef). For repeatability and reproducibility, sample were prepared in three repeats for each concentration level, resulting in a total of 18 samples for each extraction method.

#### 2.2.1. Raw Meat Extraction with Distilled Water

For both poultry (Table 2) and red meat adulteration (Table 3), 20 g of each sample (meat mixture) was extracted as described in the experiment for the determination of optimal dilution, to obtain the stock filtrates. From the stock filtrate, the pre-determined optimal dilution level was pipetted into a 100 mL volumetric flask, filled up to volume with distilled water, homogenized, and transferred into 100 mL glass beakers for e-tongue analysis.

#### 2.2.2. Meat Extraction by Cooking with Distilled Water

For both poultry (Table 2) and red meat adulteration (Table 3), 20 g of each sample was boiled in a cooking pot for 5 min 200 mL distilled water. It was then filtered with a wire mesh filter (20 mesh) to obtain the stock filtrate. From the stock filtrate, the pre-determined optimal dilution level was pipetted into a 100 mL volumetric flask, filled up to volume with distilled water, homogenized and transferred into 100 mL glass beakers for e-tongue analysis.

#### 2.2.3. Frozen Meat Extraction with Distilled Water

For both poultry (Table 2) and red meat adulteration (Table 3), 20 g of each sample was stored by freezing at a temperature −5 °C. The Frozen samples were removed on the second day of storage and put into a water bath of 50 °C for 20 min for them to defrost. Stock filtrate was then prepared from these samples as described in the method for raw meat extraction with distilled water. From the stock filtrate, the pre-determined optimal dilution level was pipetted into a 100 mL volumetric flask, filled up to volume with distilled water, homogenized, and transferred into 100 mL glass beakers for e-tongue analysis.

### 2.3. Electronic Tongue Measurements

A potentiometric electronic tongue (e-tongue) with food grade sensors (BB, HA, ZZ, GA CA, JE, JB) was used in this study and was configured according to the manufacturers (AplhaM.O.S., Toulouse, France), recommendation prior to each adulteration measurement. [41]. To configure the instrument, a conditioning was performed using 0.01 M hydrochloric acid solution and distilled water then, a calibration using the solution prepared from the extraction of pure turkey and pure beef. The purpose of this, was to achieve good sensor signals from the instrument during measurement and so as to allow rapid detection of low concentration differences in the meat mixture samples otherwise, known as fingerprinting. The main operating principle of measurement for the e-tongue is based on the difference in potential changes of sensor probes (BB, HA, ZZ, GA CA, JE, JB) against a reference electrode in zero-current conditions [20,42]. Each replicate sample from the determination of optimal dilution and the different extraction methods, was measured four times. This resulted in 12 readings in total from the e-tongue sensor for each adulterated meat mixture. The volume of each tested sample during the measurement was 100 mL, the sampling time was 120 s, the sampling frequency was 1 s, and the cleaning time with distilled water was 20 s. All experiments were performed at room temperature. Temperature correction measures [28,43] were also taken into account to compensate any temperature fluctuations.

### 2.4. Data Analysis

The average values of the last 10 s of the sensor signals, representing stabilized and optimal sensitivity of the different sensors were exported for data analysis [38]. Additive drift correction relative to the whole sample set described by Kovacs et al. [28], was applied to all the datasets for optimum sensor signals before multivariate data analysis.

Principal component analysis (PCA) was used to visual patterns in the dataset and also to detect any possible outlier before linear discriminant analysis and partial least squares regression were performed.

#### 2.4.1. Classification of Meat Mixtures with Linear Discriminant Analysis

Linear discriminant analysis (LDA) was used for multi-class classification of the different adulterated meat samples, but a process of sensor optimization was performed first. The purpose of this was to use only the sensors with the best signal data for the LDA models and it was done by running LDA simulation models in 6 steps. One sensor was removed in each step of the simulation and the average values of cross-validation were compared to the average value of cross-validation when all seven sensors were used. The sensor combination that produced the highest accuracy after cross-validation was selected and used to develop the subsequent LDA models. The sensor optimization process was only performed for the dataset from the optimal extraction method experiment. It was not done to the dataset from the optimal dilution experiment because that experiment mainly focused on determining the optimal dilution level and for fair comparison all the seven sensors were used for the model development.

**Figure 1:**
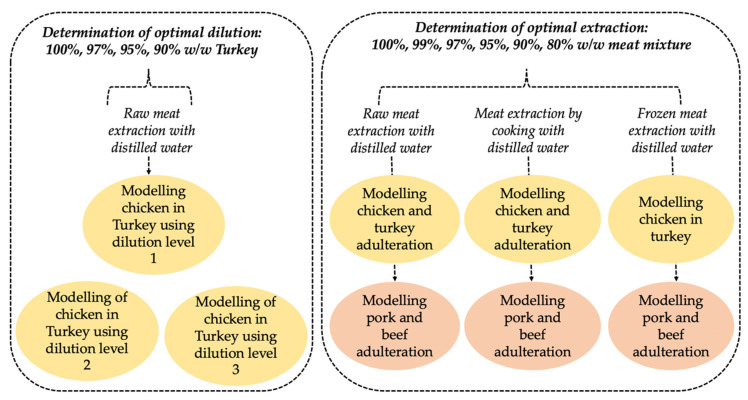
Modelling adulterated poultry and red meat with on e-tongue sensor signals.

LDA models were developed to classify the different concentrations of poultry and red meat adulteration for all the three sample preparation methods. In total, nine different LDA models were developed, as shown in Figure 1: three for the determination of optimal dilution and six for the determination of optimal extraction.

Cross validation was performed for each LDA model to evaluate their robustness in predicting meat adulteration. For this, the data was divided into a training set and a validation set. The training set was made up of two-third of the data thus, the sensor signals from the first and second replicates of each sample (meat mixture at different concentrations). The validation set was made up of sensor signals from the third replicate. The data splitting was done three times such that, each sample was used at least once in the calibration and validation set.

#### 2.4.2. Partial Least Squares Regression

Partial least squares (PLS) regression was used to develop models to regress on the concentration of chicken in turkey and on the concentration of pork in beef, but a process of sensor optimization was also performed as was done for the LDA but this time, based on the root mean square errors after cross-validation (RMSECV’s). The sensor combination that produced the lowest RMSECV was selected and used to develop the subsequent PLS regression models. The sensor optimization process was only performed for the dataset from the optimal extraction.

In all, nine different PLS regression models were developed (Figure 1): three for the determination of optimal dilution and six for the determination of optimal extraction. Cross validation was performed to evaluate the robustness of the PLSR models in predicting meat adulteration. For this, the data was again, divided into a calibration set and validation set using the same approach as that was used in the LDA. The results of PLS regression were evaluated based on the root mean square error of calibration (RMSEC) and the coefficient of determination (R^2^C); in cross-validation (RMSECV, R^2^CV). Low RMSECVs were the basis for determining the ideal latent variables in each PLS regression model.

## 3. Results

### 3.1. Determination of Optimal Dilution

#### 3.1.1. Linear Discriminant Analysis (LDA) Models Developed for the Determination of Optimal Dilution

**Figure 2:**
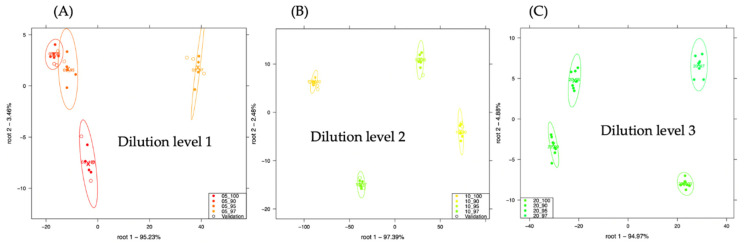
Classification of chicken and turkey adulteration using the dilution level 1 (**A**), dilution level 2 (**B**) and dilution level 3 (**C**).

Figure 2, shows the LDA model developed to classify minced chicken in turkey using all the three different dilution levels. There was a visually distinct separation patterns of the different meat samples with more than 94% of the variance expressed in the root1 of all the plots. Dilution level 2 (Figure 2B) had the most distinct separation. There was average recognition and prediction accuracy of 100% respectively for the classification of adulterated meat samples when all the three different dilution levels were used.

#### 3.1.2. Partial Least Squares (PLS) Regression Models Developed for the Determination of Optimal Dilution

All the different dilution levels produced 100% classification accuracy, so it was necessary to build PLS regression models as well to determine the optimal dilution. Table 4 shows the PLS regression models and the parameters of accuracies for predicting chicken in turkey. After cross-validation, the different adulteration levels could be predicted with coefficients of determination (R^2^CV) in the range 0.65–0.95 for all the different dilution levels. The models were also characterized by low root mean squares of cross-validation (RMSECV) generally, less than 2.14 *w/v* of turkey. The best model was achieved for dilution level 2, so this dilution level was used for subsequent experiments in the determination.

### 3.2. Optimal Extraction Method

#### 3.2.1. Results of Sensor Optimization for LDA Analysis in the Optimal Extraction Method

Table 5, shows the results of sensor optimization using all the three different sample preparation methods for chicken and turkey adulteration and pork and beef adulteration. The table column “selected sensors” represent the sensors that produced the optimized classification accuracies and, were used to develop the LDA models for the different sample preparation methods. Sensors HA, BB, ZZ, GA and JB were the most important sensors in discriminating the adulterated mixtures under study. Sensor CA was the least effective.

#### 3.2.2. LDA Analysis for Raw Meat Extraction with Distilled Water in the Determination of Optimal Extraction

**Figure 3:**
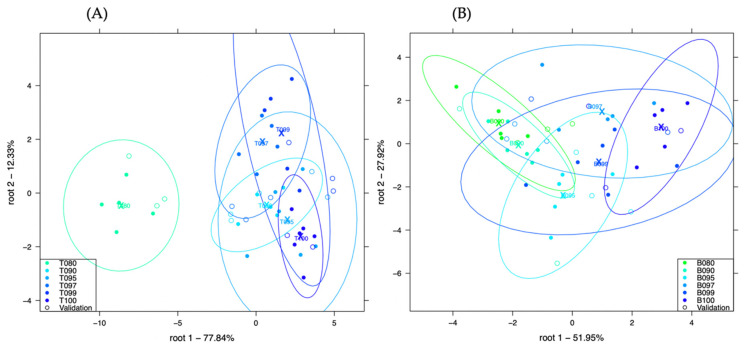
Classification of chicken and turkey adulteration (plot **A**) and pork and beef adulteration (plot **B**) using the method: raw meat extraction with distilled water.

Figure 3A, shows the LDA plot developed to classify chicken and turkey adulteration with more than 77% of the variance expressed in the root 1, whereas Figure 3B, shows the LDA plot developed to classify pork and beef adulteration with more than 51% of the variance expressed in the root 1.

Very little visual separation could be observed in the plots but primarily, sample T080 (20% *w*/*w* chicken in turkey) could be separated from the other concentrations in Figure 3B. The lower concentrations were, visually, poorly separated in both Figure 3A and Figure 3B.

Table 6, shows the confusion table for the classification of different concentrations of chicken in turkey using the method, raw meat extraction with distilled water. There was average recognition accuracy of 81.28% and prediction accuracy of 58.35%.

After cross validation, only sample T080 (20% *w*/*w* chicken) showed 100% classification, confirming the separation in the plot. Samples T090 (10% *w*/*w* chicken) and T100 (pure turkey) showed the second highest correct classification accuracies of 87.59% respectively. However, 49.81% of sample T090 was misclassified as T097 (3% *w*/*w* chicken) and 12.41% of sample T100 was misclassified as T095 (5% *w*/*w* chicken). Samples T099 (1% *w*/*w* chicken) and T097 (3% *w*/*w* chicken) had the lowest correct classification accuracies of 12.36% and 25.09% respectively.

Table 7, shows the confusion table for the classification of different concentrations of pork in beef using the method, raw meat extraction with distilled water. There was average recognition accuracy of 67.73% and prediction accuracy of 54.25%.

All the concentrations showed some misclassifications after cross-validation, confirming the poor visual separation in Figure 3B. The worst misclassification was 37.45%, observed for sample T099 (1% *w*/*w* pork). B080 (20% *w*/*w* pork) gave the best correct classification accuracy of 62.78%. Samples B095 and B097 both gave relatively good correct classification accuracies of 50% respectively.

#### 3.2.3. LDA Analysis for Meat Extraction by Cooking with Distilled Water for the Determination of Optimal Extraction

**Figure 4:**
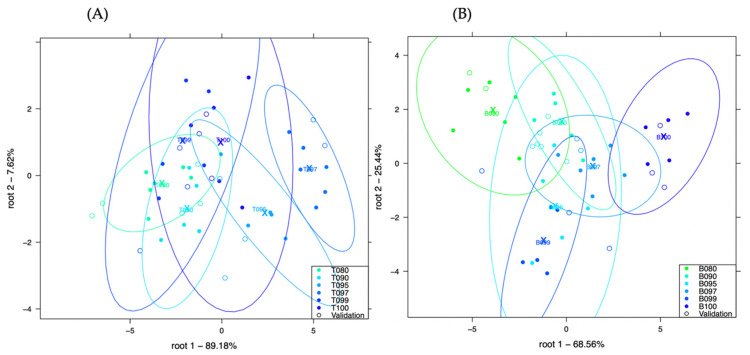
Classification of chicken and turkey adulteration (plot **A**) and pork and beef adulteration (plot **B**) using the method: method: meat extraction by cooking with distilled water.

Figure 4A, shows the LDA plot developed to classify chicken and turkey adulteration with more than 89% of the variance expressed in the root 1 whereas, Figure 4B, shows the LDA plot developed to classify pork and beef adulteration with more than 68% of the variance expressed in the root 1.

Very little visual separation could be observed in Figure 4A but primarily, samples with the lower concentrations of chicken in turkey (T095, T097 and T99) could be observed on the right side of the plot whereas, those with higher concentrations could be observed on the left. In Figure 4B, there was a decreasing pattern of mixtures of pork in beef from left to right in the plot. Samples B100 (pure beef) and B080 (20% *w*/*w* pork) could be visually distinguished.

After cross validation, sample T097 (3% *w*/*w* chicken) showed the highest classification of 87.62% classification, confirming the separation in the plot. Samples T090 (10% *w*/*w* chicken) and T080 (20% *w*/*w* chicken) also showed good correct classification accuracies of 75.19% respectively. The worst classifications were observed for T099 (1% *w*/*w* chicken) only with 25.09% correct classification.

Table 8, shows the confusion table for the classification of different concentrations of chicken in turkey using the method, meat extraction by cooking with distilled water. There was average recognition accuracy of 78.13% and prediction accuracy of 64.73%.

Table 9, shows the confusion table for the classification of different concentrations of pork in beef using the method, meat extraction by cooking with distilled water. There was average recognition accuracy of 89.62% and prediction accuracy of 68.77%.

After cross validation, only sample B100 (pure beef) showed 100% classification, confirming the separation in the plot. Samples B090 (10% *w*/*w* pork) and B080 (20% *w*/*w* pork) showed the second highest correct classification accuracies of 87.59% respectively and misclassifications of 12.41% respectively. B080 only showed misclassification with B090 whereas, B090 only showed misclassification with B095. The worst classifications were observed for B095 (5% *w*/*w* pork). B097 and B099 showed correct classification accuracies of 50% respectively.

#### 3.2.4. LDA Analysis for Frozen Meat Extraction with Distilled Water for the Determination of Optimal Extraction

**Figure 5:**
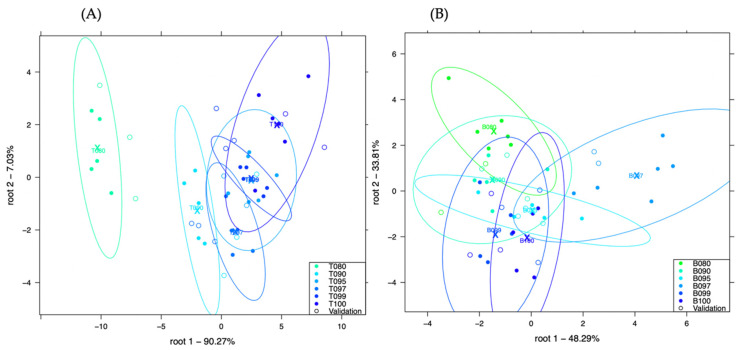
Classification of chicken and turkey adulteration (plot **A**) and pork and beef adulteration (plot **B**) using the method: frozen meat extraction with distilled water.

Figure 5A, shows the LDA plot developed to classify chicken in turkey, with more than 90% of the variance expressed in the root 1 whereas, Figure 5B, shows the LDA plot developed to classify pork in beef, with more than 48% of the variance expressed in the root 1.

Very little visual separation could be observed in Figure 5A but primarily, samples with the lower concentrations of chicken in turkey (T095, T097 and T99) could also be observed on the right side of the plot whereas, those with higher concentrations could be observed on the left. Sample T080 (20% *w*/*w* chicken) and T090 (10% *w*/*w* chicken) could be distinguished in the plot. In Figure 5B, only B080 (20% *w*/*w* pork) and B097 (3% *w*/*w* pork) showed some visual separation.

Table 10, shows the confusion table for the classification of different concentrations of chicken and turkey adulteration using the method, frozen meat extraction with distilled water. There was average recognition of 80.52% and prediction accuracy of 62.55%.

After cross validation, only sample T080 (20% *w*/*w* chicken) showed 100% classification, confirming the separation in the plot. Samples T095 (5% *w*/*w* chicken), T097 (3% *w*/*w* chicken) and T100 (pure turkey) showed the second highest correct classification accuracies of 87.62%, 93.81%, and 87.62%, respectively. The worst classification was observed for T099 (1% *w*/*w* chicken).

Table 11, shows the confusion table for the classification of different concentrations of pork and beef adulteration using the method, frozen meat extraction with distilled water. There was average recognition accuracy of 85.51% and prediction accuracy of 56.41%.

All the concentrations showed some misclassifications after cross-validation, confirming the poor visual separation in Figure 5B. The worst misclassification was 12.41%, observed for sample B099 (1% *w*/*w* pork). B097 (3% *w*/*w* pork) gave the best correct classification accuracy of 87.59%. B080 showed misclassifications with the higher ranged concentrations (B090 and B095) but not with the lower ranged ones (B097 and B099).

#### 3.2.5. Partial Least Squares (PLS) Models to Regress on the Concentrations of Adulterated Poultry and Red Meat for the Determination of Optimal Extraction

Table 12, shows the results of PLS regression sensor optimization using all the three different sample preparation methods for chicken and turkey adulteration and pork and beef adulteration. The table column “selected sensors” represent the sensors that produced the lowest root mean squared error of cross-validation (RMSECV) and, were used to develop the PLS regression models for the different sample preparation methods. Sensors HA, BB, ZZ, and GA were the most important sensors in predicting the concentration of the adulterated mixtures under study with the lowest error. Sensor CA was the least effective.

#### 3.2.6. PLS Models to Regress on the Concentrations of Chicken in Turkey Using all the Extraction Methods

Table 13, shows the PLS models to regress on adulterated poultry using the raw meat extraction with distilled water, meat extraction by cooking with distilled water and frozen meat extraction with distilled water methods. Using latent variables (LV) in the range of one to five, the different concentrations of meat samples could be predicted with R^2^CV’s higher than 0.47 and errors (RMSECV) less than 4.93 *w/v* of turkey in the samples. The best PLS model for the prediction of chicken in turkey was achieved when the frozen meat extraction with distilled water method was used.

#### 3.2.7. PLS Models to Regress on the Concentrations of Pork in Beef Using all the Extraction Methods

Table 14 shows the PLS models to regress on adulterated red meat using the raw meat extraction with distilled water, meat extraction by cooking with distilled water and frozen meat extraction with distilled water methods. Using latent variables (LV) in the range of three to five, the different concentrations of meat samples could be predicted with R^2^CV’s higher than 0.34 and errors (RMSECV) less than 5.51 *w/v* of chicken in the samples. The best PLS model for the prediction of red meat was achieved when the meat extraction by cooking method was used.

## 4. Discussion

### 4.1. Determination of Optimal Optimal Dilution

All the dilution levels used in the determination of optimal dilution, showed excellent classification accuracies of 100% in both recognition and prediction so there was a need to apply some other multivariate tools to ascertain the optimum dilution level. For this, PLS regression was performed and gave models with high R^2^CVs and low RMSECV’s in which, further confirmed the potentials of discriminating and quantifying meat adulteration with e-tongue. Dilution level 2 with 1% *w/v* chicken was proven to be the optimum dilution level because it showed the best separation of the different samples in LDA and also provided the highest R^2^CV of 0.95 and the lowest RMSECV of 0.80 *w/v* of chicken in turkey. Low RMSECVs and R^2^CVs closer to 1 represent robust models [44].

### 4.2. Determination of Optimal Extraction Method

#### 4.2.1. LDA Sensor Optimization for the Determination of Optimal Extraction Method

All the seven e-tongue sensors in the Alpha Astree liquid and taste analyzer, have often exhibited a combined effective for the discrimination and detection of food quality [45,46,47]. However, the sensors respond differently to environmental conditions that could arise during analysis and could as such influence sensor sensitivity [34]. In our case, sensors HA, BB, ZZ, GA, and JB provided the best sensor signals when LDA simulations were performed. They were the most important sensors in discriminating the adulterated meat mixtures. Sensor CA was the least effective.

#### 4.2.2. Raw Meat Extraction with Distilled Water

High misclassification rates associated with the low concentrations of meat mixtures (99%, 97% and 95%) after raw meat extraction with distilled water suggests that perhaps, the meat compounds extracted at these concentrations were not sufficient to detection and discrimination with the e-tongue. For instance, extraction of fat-soluble compounds in meat is a process often done with other methods such as the Soxhlet method, Bligh and Dyer method, Folch method, microwave solvent extraction etc. [48]. Extracting with water may, therefore, be challenging even if the fat-soluble compounds are in any case present in the meat. This is particularly true as the samples with the highest concentrations: T080 (20% *w/w* chicken) and B080 (20% *w/w* pork) always gave the best classification accuracy whereas, those with the lowest concentration T099 (1% *w*/*w* chicken) and B099 (1% *w*/*w* pork) consistently gave the worst. Extraction of meat compounds often involve using denaturing or nondenaturing solutions which, can be expensive [49]. As observed for the determination of optimal dilution, better accuracies could be achieved with this method for meat mixtures with higher adulterant ranges and when 1% *w*/*w* of mixture combination was not included. It is important to however, point out that each concentration level was prepared in three repeats for the determination of optimal extraction, but this was not done for the determination of optimal dilution as the objective was only to determine the best dilution level after sample extraction. These factors may have contributed to different forms memory effect [29] in the two experiments.

#### 4.2.3. Meat Extraction by Cooking with Distilled Water

The best average classification accuracies were generally achieved after meat extraction by cooking with distilled water for both chicken in turkey adulteration and pork in beef adulteration. With the method, the minimum correct classification of chicken in turkey was 62.36% cross-validation and even low concentrations such as T097 (3% *w*/*w* chicken) could be classified with 100% correct classification after cross-validation. This method had the best average correct classification after cross-validation in comparison to the other methods (raw meat extraction with distilled water and frozen meat extraction with distilled water). Besides time consumption, elevated temperatures have been widely acknowledge to be effective in the extraction of bioactive compounds in diverse foods [50,51] and even proved effective in the extraction of compounds from chicken bone [52]. The short cooking period of five minutes used in our study suggests that this can be a standardized meat for even other meat analysis with the e-tongue.

#### 4.2.4. Frozen Meat Extraction with Distilled Water

Like the raw meat extraction with distilled water, high misclassification rates were also exhibited using the method with frozen meat extraction with distilled water. The results suggest that, the meat compounds extracted at these concentrations using this method may not have been sufficient for detection and discrimination by the e-tongue sensors. According to literature [53], the physical and biological state of frozen and thawed meat can be influenced by factors such as the amount of water present after freezing, the temperature, rate and duration of freezing. These factors can often lead to changes in the chemical and biochemical properties of meat [54], which may have influenced the concentrations of compounds necessary for the detection and discrimination with the e-tongue sensors.

#### 4.2.5. PLS Sensor Optimization for the Determination of Optimal Extraction Method

From PLS simulations in the sensor optimization process, sensors HA, BB, ZZ and GA were the most important sensors in predicting the concentrations of the meat mixtures. These sensors provide the lowest RMSECV’s. Sensor CA was the least effective. The important and less effective sensors were also in agreement with those obtained from LDA analysis.

#### 4.2.6. PLS Regression Models for Predicting Concentrations of Chicken in Turkey and Pork in Beef Using all the Three Extraction Methods

PLSR results for predicting the concentrations of pork in beef confirmed the accuracies achieved for the LDA analysis. The extraction method by cooking with distilled water gave the best accuracies in comparison to the other two methods. This was, however, contrary to the PLSR results for predicting the concentrations of chicken in turkey where, the extraction by cooking with distilled water method gave the worst accuracy. The best accuracy was achieved with the frozen meat extraction with distilled water.

## 5. Conclusions

LDA and PLSR results from the optimal dilution experiment where, lower concentration ranges of chicken and turkey adulteration were used, showed that, the dilution level 2 (10 mL of extract in 100 mL distilled water) was the optimum for e-tongue analysis. Based on this, other experiments were performed with wider concentration ranges poultry (chicken and turkey) and red meat (pork and beef) meat samples using three different extraction methods. The dataset from all the different extraction methods were drift corrected according to literature and although a novel step of sensor optimization improved the accuracies of analysis, the memory effect of the e-tongue was still suspected to contribute to some of the misclassifications in the study as observed other e-tongue experiments. All three extraction methods, however, produced good average recognition accuracies higher than 78% and average prediction accuracies higher than 56% which, are comparable with the accuracies obtained with other methods such as near infrared spectroscopy. Concentrations of 1% *w*/*w* of pork and 1% *w*/*w* chicken, were the most difficult to classify after cross-validation using all the different extraction methods. Comparing the different extraction methods, the raw meat extraction with distilled water and frozen meat extraction with distilled water gave the worst average prediction accuracies for discriminating chicken in turkey and red meat pork in beef probably due to the presence of fat-soluble compounds which can inhibit water extraction methods as proven in literature. The best average prediction accuracies were achieved with the method of meat extraction by cooking with distilled water. Sensors HA, BB, ZZ, GA, and JB were the most important sensors in discriminating the adulterated meat mixtures. Sensor CA was the least effective. Our findings provide grounds for future application of the e-tongue for meat analysis but studies with larger datasets are recommended for more defined limits of detection that can be adopted by regulatory authorities. Sensor signals from the e-tongue can also be corelated with reference data from other conventional analytical methods for the rapid prediction of meat properties, which is cost effective as most conventional methods require reagents and waste management tools.

## Figures and Tables

**Table 1:** Mixture combination for turkey and chicken adulteration for determination of optimal dilution.

Sample ID	Turkey (% *w*/*w*)	Chicken (% *w*/*w*)	Turkey (g)	Chicken (g)	% *w*/*v* of Meat Mixture
05_100	100	0	20.00	0.00	0.50
05_097	97	3	19.40	0.60	0.50
05_095	95	5	19.00	1.00	0.50
05_090	90	10	18.00	2.00	0.50
10_100	100	0	20.00	0.00	1.00
10_097	97	3	19.40	0.60	1.00
10_095	95	5	19.00	1.00	1.00
10_090	90	10	18.00	2.00	1.00
20_100	100	0	20.00	0.00	2.00
20_097	97	3	19.40	0.60	2.00
20_095	95	5	19.00	1.00	2.00
20_090	90	10	18.00	2.00	2.00

**Table 2:** Mixture combination for turkey and chicken adulteration for determination of optimal extraction.

Sample ID	Turkey (%)	Chicken (%)	Turkey (g)	Chicken (g)
T100	100	0	20.00	0.00
T099	99	1	19.80	0.20
T097	97	3	19.40	0.60
T095	95	5	19.00	1.00
T090	90	10	18.00	2.00
T080	80	20	16.00	4.00

**Table 3:** Mixture combination for beef and pork adulteration determination of optimal extraction.

Sample ID	Beef (%)	Pork (%)	Beef (g)	Pork (g)
B100	100	0	20.00	0.00
B099	99	1	19.80	0.20
B097	97	3	19.40	0.60
B095	95	5	19.00	1.00
B090	90	10	18.00	2.00
B080	80	20	16.00	4.00

**Table 4:** PLS models to regress the concentration of turkey in turkey/chicken meat mixtures extracted and diluted with three different dilution levels and drift corrected dataset.

Dilution Level	LV	R^2^	RMSEC (*w*/*v* Meat Mixture)	R^2^CV	RMSECV (*w*/*v* Meat Mixture)
Dilution level 1 (2% *w/v* turkey)	3	0.88	1.26	0.81	1.57
Dilution level 2 (1% *w/v* turkey)	3	0.97	0.59	0.95	0.80
Dilution level 3 (0.5% *w/v* turkey)	1	0.71	1.96	0.65	2.14

**Table 5:** Results of LDA sensor optimization using all the three different sample preparation methods for chicken and turkey adulteration and pork and beef adulteration.

Meat Combination	Sample Preparation	Selected Sensors	Omitted Sensors	Initial Cross-Validation Accuracies (%)	Optimized Cross-Validation Accuracies (%)
Chicken and turkey adulteration	Raw meat extraction with distilled water	HA, BB, ZZ, GA	JE, CA, JB	47.99	58.35
	Meat extraction by cooking with distilled water	BB, ZZ, GA, JB	HA, JE, CA	54.14	64.72
	Frozen meat extraction with distilled water	All: HA, BB, ZZ, GA, JE, JB, CA	None	62.55	62.55
Pork and beef adulteration	Raw meat extraction with distilled water	HA, ZZ, GA, JB	BB, CA, JE	45.90	54.25
	Meat extraction by cooking with distilled water	HA, ZZ, GA CA, JE, JB	BB	58.37	68.77
	Frozen meat extraction with distilled water	HA, ZZ, BB, GA, JE, JB	CA	52.11	56.41

**Table 6:** Confusion table for the classification of chicken in turkey using the method: raw meat extraction with distilled water. Classifications are expressed as percentages (%).

Average Accuracies		T080	T090	T095	T097	T099	T100
Recognition 81.28%	T080	100	0	0	0	0	0
	T090	0	93.81	18.73	24.95	0	0
	T095	0	0	56.18	0	12.57	6.19
	T097	0	6.19	0	68.86	6.19	0
	T099	0	0	12.55	6.19	75.05	0
	T100	0	0	12.55	0	6.19	93.81
Cross-validation 58.35%	T080	100	0	0	0	0	0
	T090	0	87.59	0	49.81	0	0
	T095	0	0	37.45	0	25.09	12.41
	T097	0	12.41	12.36	25.09	37.45	0
	T099	0	0	25.09	25.09	12.36	0
	T100	0	0	25.09	0	25.09	87.59

**Table 7:** Confusion table for the classification of different concentrations of pork in beef using the method: raw meat extraction with distilled water. Classifications are expressed as percentages (%).

Average Accuracies		B080	B090	B095	B097	B099	B100
Recognition 67.73%	B080	75.05	6.19	0	24.95	12.57	0
	B090	6.19	75.05	0	0	0	0
	B095	0	12.57	75.05	0	18.76	0
	B097	18.76	6.19	6.19	56.29	6.19	0
	B099	0	0	18.76	12.57	43.71	18.76
	B100	0	0	0	6.19	18.76	81.24
Cross-validation 54.25%	B080	62.78	12.41	0	25.19	25.09	0
	B090	12.41	62.78	0	12.41	0	0
	B095	0	12.41	50	0	12.36	0
	B097	12.41	12.41	12.41	50	0	0
	B099	12.41	0	37.59	0	37.45	37.45
	B100	0	0	0	12.41	25.09	62.55

**Table 8:** Confusion table for the classification of different concentrations of chicken and turkey adulteration using the method: meat extraction by cooking with distilled water. Classifications are expressed as percentages (%).

Average Accuracies		T080	T090	T095	T097	T099	T100
Recognition 78.13%	T080	74.91	0	0	0	6.19	0
	T090	12.55	87.62	0	0	6.19	12.55
	T095	0	0	75.05	0	0	12.55
	T097	0	0	0	100	0	0
	T099	12.55	6.19	0	0	68.86	12.55
	T100	0	6.19	24.95	0	18.76	62.36
Cross-validation 64.72%	T080	75.19	0	0	0	12.36	0
	T090	12.41	75.19	0	0	25.09	12.41
	T095	0	0	62.55	12.41	0	12.41
	T097	0	0	12.36	87.59	0	0
	T099	12.41	12.41	0	0	25.09	12.41
	T100	0	12.41	25.09	0	37.45	62.78

**Table 9:** Confusion table for the classification of different concentrations of pork and beef adulteration using the method: meat extraction by cooking with distilled water. Classifications are expressed as percentages (%).

Average Accuracies		B080	B090	B095	B097	B099	B100
Recognition 89.62%	B080	100	0	0	0	0	0
	B090	0	87.62	18.73	6.19	0	0
	B095	0	6.19	68.73	6.19	6.19	0
	B097	0	6.19	0	87.62	0	0
	B099	0	0	12.55	0	93.81	0
	B100	0	0	0	0	0	100
Cross-validation 68.77%	B080	87.59	0	0	0	12.41	0
	B090	12.41	87.59	25.09	12.41	0	0
	B095	0	12.41	37.45	25.19	25.19	0
	B097	0	0	0	50	12.41	0
	B099	0	0	37.45	12.41	50	0
	B100	0	0	0	0	0	100

**Table 10:** Confusion table for the classification of different concentrations of chicken and turkey adulteration using the method: frozen meat extraction with distilled water. Classifications are expressed as percentages (%).

Average Accuracies		T080	T090	T095	T097	T099	T100
Recognition 80.52%	T080	100	0	0	0	0	0
	T090	0	81.39	6.19	6.19	0	0
	T095	0	6.2	87.62	0	18.76	0
	T097	0	6.2	0	93.81	0	0
	T099	0	6.2	6.19	0	81.24	12.55
	T100	0	0	0	0	0	87.45
Cross-validation 62.55%	T080	100	0	0	0	0	0
	T090	0	37.45	0	25.09	0	0
	T095	0	25.09	62.55	12.36	50	12.41
	T097	0	25.09	0	62.55	12.41	0
	T099	0	12.36	25.09	0	37.59	12.41
	T100	0	0	12.36	0	0	75.19

**Table 11:** Confusion table for the classification of different concentrations of pork and beef adulteration using the method: frozen meat extraction with distilled water. Classifications are expressed as percentages (%).

Average Accuracies		B080	B090	B095	B097	B099	B100
Recognition 85.51%	B080	100	0	0	0	0	0
	B090	0	93.81	12.55	0	6.19	0
	B095	0	0	68.73	0	6.19	12.55
	B097	0	0	6.18	100	0	0
	B099	0	6.19	12.55	0	75.05	12.55
	B100	0	0	0	0	12.57	74.91
Cross-validation 56.41%	B080	50	0	0	0	0	0
	B090	37.59	100	12.41	0	12.41	0
	B095	12.41	0	62.78	12.41	25.19	37.45
	B097	0	0	0	87.59	0	0
	B099	0	0	12.41	0	12.41	37.45
	B100	0	0	12.41	0	50	25.09

**Table 12:** Results of PLS regression sensor optimization using all the three different extraction methods for chicken and turkey adulteration and pork and beef adulteration.

Meat Combination	Sample Preparation	Selected Sensors	Omitted Sensors	Initial RMSECV (% *w*/*v* Meat Mixture)	Optimized RMSECV (% *w*/*v* Meat Mixture)
Turkey and chicken adulteration	Raw meat extraction with distilled water	HA, BB, ZZ, GA,	JE, JB, CA	3.68	3.34
	Meat extraction by cooking with distilled water	HA, BB, ZZ, CA, JB	JE, GA	5.19	4.93
	Frozen meat extraction with distilled water	HA, BB, ZZ, GA, JE	JB, CA	3.04	2.89
Beef and beef pork adulteration	Raw meat extraction with distilled water	HA, BB, CA, GA	JE, JB, ZZ	5.91	5.51
	Meat extraction by cooking with distilled water	HA, ZZ, JB	JE, GA, BB, CA	4.44	3.83
	Frozen meat extraction with distilled water	HA, BB, ZZ JB, JE	GA, CA	5.81	5.16

**Table 13:** PLS models to regress the concentration of chicken in turkey using all the three extraction methods.

Sample Preparation Method	LV	R^2^	RMSEC (*w*/*v* Meat Mixture)	R^2^CV	RMSECV (*w*/*v* Meat Mixture)
Raw meat extraction with distilled water	3	0.82	2.91	0.76	3.34
Meat extraction by cooking with distilled water	5	0.67	3.92	0.47	4.93
Frozen meat extraction with distilled water	4	0.86	2.57	0.81	2.89

**Table 14:** PLS models to regress the concentration of pork in beef using all the three extraction methods.

Sample Preparation Method	LV	R^2^	RMSEC (*w*/*v* Meat Mixture)	R^2^CV	RMSECV (*w*/*v* Meat Mixture)
Raw meat extraction with distilled water	4	0.51	4.78	0.34	5.51
Meat extraction by cooking with distilled water	3	0.76	3.35	0.72	3.83
Frozen meat extraction with distilled water	4	0.65	4.05	0.43	5.16

## Data Availability

The data presented in this study are available on request from the corresponding author. The data are not publicly available due to privacy and ethical reasons.
